# Quantitative Label-Free Comparison of the Metabolic Protein Fraction in Old and Modern Italian Wheat Genotypes by a Shotgun Approach

**DOI:** 10.3390/molecules26092596

**Published:** 2021-04-29

**Authors:** Antonella Di Francesco, Vincenzo Cunsolo, Rosaria Saletti, Birte Svensson, Vera Muccilli, Pasquale De Vita, Salvatore Foti

**Affiliations:** 1 Laboratory of Organic Mass Spectrometry, Department of Chemical Sciences, University of Catania, Viale A. Doria 6, 95125 Catania, Italy; antonelladfrancesco@gmail.com (A.D.F.); rsaletti@unict.it (R.S.); v.muccilli@unict.it (V.M.); sfoti@unict.it (S.F.); 2 Department of Biotechnology and Bioengineering, Technical University of Denmark, Søltofts Plads, Building 224, 2800 Kgs. Lyngby, Denmark; bis@bio.dtu.dk; 3 CREA Research Centre for Cereal and Industrial Crops (CREA-CI), S.S. 673 km 25.200, 71122 Foggia, Italy; pasquale.devita@crea.gov.it

**Keywords:** old and modern wheat genotypes, label-free quantitation, high-resolution mass spectrometry, proteome analysis, metabolic proteins

## Abstract

Wheat represents one of the most important cereals for mankind. However, since wheat proteins are also the causative agent of several adverse reactions, during the last decades, consumers have shown an increasing interest in the old wheat genotypes, which are generally perceived as more “natural” and healthier than the modern ones. Comparison of nutritional value for modern and old wheat genotypes is still controversial, and to evaluate the real impact of these foods on human health comparative experiments involving old and modern genotypes are desirable. The nutritional quality of grain is correlated with its proteomic composition that depends on the interplay between the genetic characteristics of the plant and external factors related to the environment. We report here the label-free shotgun quantitative comparison of the metabolic protein fractions of two old Sicilian landraces (Russello and Timilia) and the modern variety Simeto, from the 2010–2011 and 2011–2012 growing seasons. The overall results show that Timilia presents the major differences with respect to the other two genotypes investigated. These differences may be related to different defense mechanisms and some other peculiar properties of these genotypes. On the other hand, our results confirm previous results leading to the conclusion that with respect to a nutritional value evaluation, there is a substantial equivalence between old and modern wheat genotypes. Data are available via ProteomeXchange with identifier <PXD024204>.

## 1. Introduction

Wheat (*Triticum aestivum* L. ssp. *aestivum*, or “soft” wheat and *Triticum turgidum* L. ssp. *durum*, or “durum” wheat) is undoubtedly the most consumed crop in the world, making substantial contributions to the dietary intake of energy and the consequent impact on human health. Wheat proteins are not only the main source of nutritional and sensory properties but also main responsible for the technological performance of dough in relation to pasta-making. Consequently, they have been extensively investigated, mostly by MS-based methods [1,2,3]. These studies range from the characterization of the sequence of kernel proteins [4,5,6,7] to comparative proteomics investigations of different cultivars [8], and of glutenin proteins responsive to drought and low-temperature stress [9], including translocated or transgenic wheat varieties [10,11,12]. Despite their important role for human nutrition, wheat proteins are also the causative agent of celiac disease (CD) [13] and other adverse reactions, such as immunoglobulin E (IgE) mediated allergies and a less well-defined condition classified as non-celiac wheat sensitivity (NCWS) [14,15,16]. Therefore, in recent years, consumers have shown a growing interest in gluten-free foodstuffs, and in the old wheat genotypes, which are generally perceived as more “natural” and healthier than the modern ones. Even if there is no accurate clarification, it is usually accepted that old wheat genotypes have remained unchanged over the last hundred years [17]. By contrast, modern wheat includes genotypes generated in the second half of the 20th century, during the so-called “Green-Revolution” [18]. The most common commercially available old wheat species are einkorn (*Triticum monococcum* L. ssp. *monococcum*), emmer (*T. turgidum* L. ssp. *dicoccum*), khorasan (*T. turgidum* ssp. *turanicum*) and spelt (*T. aestivum* L. ssp. *spelta*). Additionally, there are several old genotypes of both *T. aestivum* and *T. durum* cultivated from the mid-1800s to the beginning of the 20th century (before the “Green Revolution”) including landraces of durum wheat, such as Russello, Senatore Cappelli, Timilia or Tumminia, and “soft” wheat, such as Gentil Rosso, Maiorca, Sieve, Solina, and Verna. Comparison of the nutritional value of modern and old wheat genotypes is still controversial, suggesting that further studies are desirable [19]. Comparative analysis of proteins, currently ongoing with a particular focus on gluten components, indicates that the wheat breeding activity carried out during the 20th century apparently improved the durum wheat gluten quality in relation to technological performance, without aggravating the allergenic potential and the content of potentially toxic immune-stimulating peptides [20,21,22]. However, a comparison of the metabolic protein fractions (salt-soluble proteins) in old and modern durum wheat genotypes is still scant. Recently, a qualitative comparison [23] was performed of the metabolic and Chloroform-Methanol (CM)-like protein fractions from two old Sicilian landraces (Russello and Timilia Reste Bianche) and an improved modern durum wheat variety (Simeto). This study revealed a remarkable qualitative similarity between old and modern genotypes, leading to the conclusion that with respect to food and nutritional value, there is a substantial equivalence among these cultivars.

In the present work, we have extended the comparison by applying a label-free proteomics approach to detect differentially abundant proteins (DAPs) between the two old Sicilian landraces Russello and Timilia Reste Bianche (hereinafter called Timilia), and the modern genotype Simeto. This study may contribute to improving the understanding of the relationship between protein profile and relative abundancy in old wheat genotypes, their potential benefits for human consumption, and phenotyping properties.

## 2. Results

A quantitative comparison using a label-free strategy of the metabolic protein fractions of two old Sicilian landraces (Russello and Timilia) and a modern genotype (Simeto) was performed for each genotype from two growing seasons (2010–2011 and 2011–2012) and three biological replicates for each season. Moreover, to assess the reproducibility of MS data, each biological replicate was subjected to triplicate RP-nHPLC/nESI-MS/MS analyses, giving rise to eighteen runs for each genotype (nine analyses for each season). To detect proteins whose abundance is depending on the growing season (Differential Abundant Proteins, DAPs, hereinafter called intra-genotype DAPs), each genotype was investigated by a quantitative comparison between the two growing seasons 2010–2011 and 2011–2012. The season 2011–2012 was chosen as a reference. To identify DAPs among the three genotypes (hereinafter called inter-genotype DAPs), a pairwise comparison for both growing seasons was carried out. In the pairwise comparison of the two old genotypes with Simeto, the modern cultivar was chosen as a reference. In the pairwise comparison between the old cultivars, Russello was selected as a reference. The lists of intra- and inter-genotype DAPs are reported and commented in the Supplementary Materials and Tables S1 and S2, respectively. Briefly, each genotype revealed some intra-genotype DAPs (fold-change <0.5 or >2.0) between the two growing seasons. The list of intra-genotype DAPs is reported in Table S1. Similarly, the pairwise inter-genotype comparison revealed a lot of inter-genotypes DAPs (fold-change <0.5 or >2.0; Table S2). Several inter-genotypes DAPs are shared among the biological replicates of the two growing seasons, suggesting that these DAPs may be related to the wheat genotype. On the other hand, some inter-genotype DAPs appear up- or down-regulated only in one out of two seasons (Table S2). Therefore, the different abundance of the latter cannot be related to the wheat genotype, but rather may depend on the growing season.

### Differentially Abundant Proteins Genotype-Related

Table 1 reports the corresponding protein profile heat map, whereas Table 2 shows the inter-genotypes DAPs revealed in both the growing seasons investigated. Three proteins appear differentially abundant in the comparison Russello vs. Simeto, whereas nine and eight DAPs were revealed in the comparisons Timilia vs. Simeto, and Timilia vs. Russello, respectively. In particular, the comparison of Russello vs. Simeto reveals that the avenin-like b1 and serpin-Z1C are down-regulated in Russello, whereas the oil body-associated protein 1A is up-regulated. The group of DAPs in the comparison Timilia vs. Simeto includes two down-regulated components in the old genotype (26 kDa endochitinase 2 and antifungal protein R) and seven up-regulated (α-amylase inhibitor 0.28, avenin-like b1, L-ascorbate peroxidase 2, oil body-associated protein 1A, subtilisin-chymotrypsin inhibitor WSCI, sucrose synthase 1, and sucrose synthase 3). Finally, the comparison Timilia vs. Russello shows three down-regulated proteins in Timilia (i.e., 26 kDa endochitinase 2, α-amylase inhibitor WDAI-3, and serpin-Z2A) and five up-regulated ones (avenin-like b1, linoleate 9S-lipoxygenase 1, subtilisin-chymotrypsin inhibitor WSCI, sucrose synthase 1, and sucrose synthase 3). 

**Figure 1:**
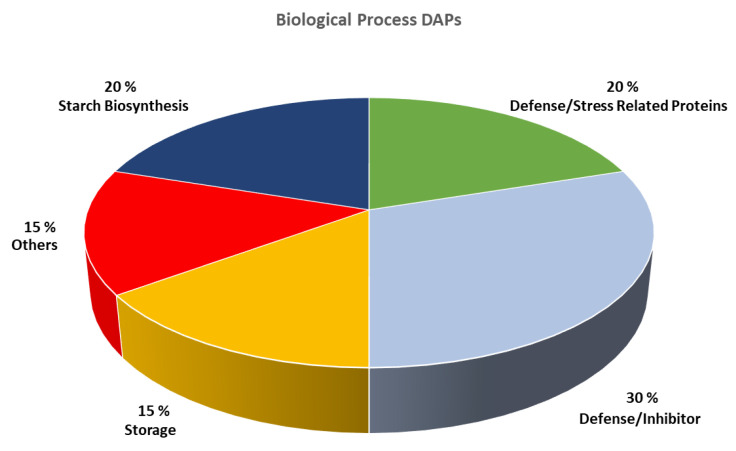
Pie chart showing the distribution of biological processes in which are involved the differential abundant proteins observed across the investigated genotypes.

Figure 1 shows the distribution of the biological processes in which are involved the inter-genotypes DAPs. Most of the DAPs are play a role in the defense mechanism of the plant because 30% acts as inhibitors, and 20% are stress-related proteins; 15% are storage proteins, and 20% proteins are involved in starch biosynthesis. Finally, the remaining 15% of the DAPs are involved in other biological processes.

It is interesting to note that, some of the proteins reported in Table 2 present similar or very reproducible values of fold-change in the seasons investigated, while others show significant variations. In this respect, there are some important aspects worth elaborating upon. First of all, it can be noted that four proteins (i.e., serpin-Z1C, avenin-like b1, L-ascorbate peroxidase 2 cytosolic, and α-amylase inhibitor WDAI-3) are also intra-genotypes DAPs (see Table S1), as they are up-regulated in the season 2010–2011 for the genotypes Russello (serpin-Z1C) and Timilia (avenin-like b1, L-ascorbate peroxidase 2 cytosolic, and α-amylase inhibitor WDAI-3). Therefore, these DAPs may be considered both genotype- and growing season-related.

## 3. Discussion

In recent years, gluten proteins have attracted much attention because of their importance in determining the functional properties of wheat flour doughs and their roles in human health. In the present study, a label-free shotgun approach was used to quantify the metabolic proteins extracted in two different growing seasons from three wheat genotypes: two old landraces Russello and Timilia, and the modern cultivar Simeto. Environmental variables such as temperature, water, and fertilizer influence the rate and duration of wheat grain development, protein accumulation, and starch deposition in unique ways, and by different mechanisms [24]. Indeed, cross-comparison of the DAPs observed among our selected genotypes, and for each genotype between the two growing seasons, revealed that the abundance of many proteins is season-related. The results indicated that a non-crossover interaction (scale effects) was involved in genotype × growing seasons interaction for all proteins detected as DAPs, since the mean expression level recorded in the first season investigated (2010–2011) was always higher than in the second (Table S1), probably due to the different weather conditions. Compared to the second year, taken as a reference in the present study, the first growing season was characterized by heavy rainfall (627.4 vs. 344.4 mm, respectively) and a lower mean temperature (12.1 vs. 12.5 °C, respectively).

Notably, a group of DAPs that appear genotype-related was also detected. Focusing the discussion on the DAPs related to the genotype, these comparisons revealed few but interesting differences between the old genotypes and the modern one, but also between the two old genotypes, Russello and Timilia, which despite being two local Sicilian varieties and both adapted to the extreme Mediterranean environments [25], are genetically differentiated as showed by Taranto et al. [26]. In their study, analyzing a large collection of genetic materials including landraces, old and modern durum wheat varieties, all accessions of Timilia grouped in a separate cluster showing a high genetic distance with most of the accessions analyzed and also with Russello. This suggested that the genetic base of Timilia was unique and it was probably associated with the peculiar morpho-phenological and grain quality traits [27,28,29] that made it particularly suitable for spring sowing in Sicily [30]. The proteome analysis carried out in the present study supported this hypothesis since Timilia presented significant differences compared to both the modern variety Simeto and the old Russello variety. While in the first case the behavior was expected [27], the result of the second case is quite noteworthy and confirms the different origin of the Timilia variety compared to the other local Sicilian durum wheat populations (i.e., Russello). 

It is interesting to note that some of the DAPs related to the genotype were also identified as intra-genotype DAPs for the old genotypes Russello and Timilia (see Table 2 and Table S1). Therefore, these proteins may be also considered as growing season-related and might represent specific targets for future investigations aimed to modulate the abundance of these proteins in relation to the growing conditions. 

Moreover, the distribution of the biological processes in which are involved the inter-genotypes DAPs reveals some interesting aspects. Indeed, if we consider that the two old durum wheat landraces were extensively cultivated for centuries under extreme environmental conditions, it is not surprising to observe how 50% of the DAPs are involved in the defense mechanism of the plant (as stress-related proteins or inhibitors), but also storage proteins (15%) and proteins involved in starch biosynthesis (20%) are among the DAPs (Figure 1). 

In particular, Timilia represented the genotype for which adaptation traits, particularly those that increase the duration of photosynthesis and the disease resistances, have been conserved. Concerning the DAPs involved in the defense mechanism, some interesting differences were detected. In particular, a significant up-regulation of the subtilisin chymotrypsin inhibitor (WSCI) was observed in Timilia in comparison with Simeto and Russello. Moreover, a down-regulation of the serpin Z2A was detected in Timilia in comparison with Russello. Finally, the serpin Z1C appears down-regulated in the old genotype Russello in comparison with the modern Simeto. WSCI and serpins are involved in the defense of the plant against insects and pathogens. Thus, they may represent potential targets to improve the disease resistance in wheat [31,32,33,34].

Serpins, which are essential for plant growth, development, and responses to stress [35], also represent allergenic wheat proteins [36,37] because they are the causative agents of cutaneous, gastrointestinal, and respiratory symptoms such as the baker’s asthma [38,39,40]. Another class of proteins, the α-amylase inhibitors, also related to the baker’s asthma, [37,41] showed a different level of abundance. In particular, the α-amylase inhibitor WDAI-3 is down-regulated in Timilia in comparison with Russello, while the α-amylase inhibitor 0.28 appears the most up-regulated protein (fold-change 64.00) in the genotype Timilia in comparison with Simeto (Table 2). Regarding the α-amylase inhibitor 0.28, it is interesting to note that this protein was not detected in the protein extracts of the season 2010–2011 of the genotype Russello, whereas in the season 2011–2012 it is strongly down-regulated in Russello in comparison with both Simeto (Russello vs. Simeto, fold-change 0.17; Table S2a) and Timilia (Timilia vs. Russello, fold-change 64.00; Table S2c). Taking into account these results, it can be deduced that the genotype Timilia presents a very high relative abundance of α-amylase inhibitor 0.28 in comparison with the other two genotypes here investigated.

Additionally, the genotype Timilia presents three stress-related proteins that show a different level of expression compared to the other two genotypes. In detail, the L-ascorbate peroxidase 2 cytosolic is up-regulated in comparison with the modern genotype Simeto. Instead, an antifungal R protein and the 26 kDa endochitinase 2 appear down-regulated in comparison with Simeto and Russello, respectively. These proteins are usually up-regulated under fungal (e.g., *Aspergillus parasiticus* or *A. flavus*) attacks accompanied by drought stress and might contribute to increase the resistance of the plant to adverse biotic and abiotic stimuli [42,43]. Altogether, the differences observed in the abundance of both the inhibitors and stress-related proteins suggest that the old genotypes, and in particular Timilia, in comparison with the modern Simeto might present different mechanisms of defense. 

Another interesting result concerns two key enzymes involved in starch biosynthesis, the major determinant of flour quality. These DAPs are the SUS1 and SUS3, up-regulated in Timilia in comparison with the other two cultivars. In this respect, it has been reported that high expression levels of sucrose synthase may indirectly contribute to a relatively high efficiency of starch biosynthesis [35]. Hou et al. [44] showed that the endosperm starch synthesis pathway was a major target of indirect selection in global wheat breeding for high yield, therefore the different expression in Timilia compared to the other two varieties could be the consequence of natural selection for extreme environmental conditions.

Timilia is also the genotype with the highest amount of the avenin-like b1 protein, whereas Russello shows the lower amount. Avenin-like proteins (ALPs) are storage proteins considered as atypical gluten constituents with positive effects on dough properties [45]. In particular, it has been demonstrated that the over-expression of avenin-like b proteins plays a positive role in improving the flour mixing characteristics, significantly enhancing the dough elasticity, resistance to extension, and stability [46]. In this respect, the avenin-like b protein could be an excellent candidate to improve the functional properties of wheat because it can be incorporated into the gluten polymers by inter-chain disulfide bonds [47]. Then, the different expression of avenin-like b1 could be responsible for quality differences, concerning gluten and dough quality, between Timilia and the other two cultivars [17].

Recently, Zhang et al. [48] also showed a clear difference in expression levels for transcripts encoding ALPs in the starchy endosperm between *Chinese Spring* and two other bread wheat varieties. In their study, a clear effect of temperatures was observed on the expression level of transcripts encoding ALPs, chitinases, glutathione S-transferases, serine carboxypeptidases and peroxidases, which confirms our results observed for the two growing seasons.

Moreover, the comparison between the two old genotypes shows the up-regulation of the Linoleate 9S-lipoxygenase 1 in Timilia. Linoleate lipoxygenase (LOX1) is a class of non-heme iron-containing dioxygenases involved in lipid oxidation. In plants, products of the LOX reaction have been shown to have roles in several processes, such as vegetative growth, wounding, response to herbivore and pathogen attack, and also in the mobilization of storage lipids during germination [49]. In durum wheat semolina, radicals produced during the intermediate states of linoleate hydroperoxidation can cause oxidative degradation of carotenoid pigments, which are mainly responsible for the yellow color of pasta products, an important parameter in the definition of pasta quality. Our findings, in agreement with Verlotta et al. [50], confirmed different Lpx-B1 expression profiles and LOX activity in mature grains among the varieties investigated in the present study since Timilia was characterized by higher levels of LOX1 protein compared to Russello, in both growing seasons. 

Finally, both the old genotypes show a significant up-regulation of the oil body-associated protein 1A—a protein involved in the maintaining of the structure of special cytoplasmatic organelles of the plant called oil body—and play an important role in seed germination [51]. 

In conclusion, continued advances in analytical techniques and genomics will help reveal the exact role of all DAPs identified in this study and possibly provide new opportunities to increase stress resistance, improve yield and grain quality of new durum wheat varieties. Of course, among the varieties analyzed, Timilia certainly represents a genetic material of interest that could be exploited for the introgression of useful alleles in modern cultivars, as they were used to a limited extent, unlike Russello and other landraces [26].

## 4. Materials and Methods

### 4.1. Chemicals

All chemicals were of the highest purity commercially available and used without further purification. KCl, K_2_HPO_4_, acetic acid and Tris-HCl were purchased from Carlo Erba (Milan, Italy). Formic Acid (FA), Protease Inhibitor Cocktail, EDTA, ammonium bicarbonate, dithiothreitol (DTT) and iodoacetamide (IAA) were obtained from Aldrich (St. Louis. MI, USA). Modified porcine trypsin was purchased from Promega (Madison, WI, USA). Water and acetonitrile (ACN) (OPTIMA^®^ LC/MS grade) for LC/MS analyses were purchased from Fisher Scientific (Milan, Italy). 

### 4.2. Samples Collection and Treatment

Two old Sicilian durum wheat landraces, Russello (released in 1910, a selection from landrace “Russie”) and Timilia Reste Bianche (1900, indigenous landrace population from Sicily), were chosen for the analysis. Simeto (1988), an improved durum wheat variety widespread in Italy and other Mediterranean countries, was chosen as representative of the most widespread commercial cultivars.

Three biological replicates of Russello, Timilia, and Simeto were provided from the Cereal Research Centre (CREA) of Foggia (Italy). The genetic materials were sowed at Foggia, during the 2010–2011 and 2011–2012 growing seasons, following a randomized block design with three replicates for each season. Grain samples were harvested, and the flours were stored at 4 °C. Wheat flour (200 mg) were suspended in 2 mL cold (4 °C) extraction solution (50 mM Tris-HCl, 100 mM KCl, 5 mM EDTA, Protease Inhibitor Cocktail, pH 7.8). The solution was incubated on ice (5 min) with intermittent mixing and centrifuged (13,523× *g*, 15 min, 4 °C). The supernatants from these extractions were stored at −80 °C until required. The concentration for each extract was determined by a fluorimetric assay using the Qubit Protein Assay kit with the Qubit 1.0 Fluorometer (ThermoFisher Scientific, Milan, Italy). Chicken lysozyme C (0.8 µg) was added as an internal standard to 50 µg (about 50 µL) of each protein extract. Finally, 50 µg of each sample was reduced adding 39 µg of DTT (3 h, 20 °C), alkylated with 94 µg of IAA (1 h, in the dark at 20 °C) and digested by porcine trypsin (Sequencing Grade Modified Trypsin Porcine, lyophilized, Promega) at an enzyme-substrate ratio of 1:50 (overnight, 37 °C). To obtain a final concentration of 25 ng/µL for each sample, and 0.4 ng/µL for Chicken lysozyme, a 5% aqueous solution of formic acid was added to obtain a final volume of 2 mL.

### 4.3. Mass Spectrometry Analysis

Mass spectrometry data were acquired on a Thermo Fisher Scientific Orbitrap Fusion Tribrid^®^ (Q-OT-qIT) mass spectrometer (Thermo Fisher Scientific, Bremen, Germany). Liquid chromatography was carried out using a Thermo Scientific Dionex UltiMate 3000 RSLC nano system (Sunnyvale, CA, USA). One microliter of peptide mixture was loaded onto an Acclaim ^®^Nano Trap C18 Column (100 µm i.d. × 2 cm, 5 µm particle size, 100 Å). After washing the trapping column with solvent A (H_2_O + 0.1% FA) for 3 min at a flow rate of 7 μL/min, the peptides were eluted from the trapping column onto a PepMap^®^ RSLC C18 EASY-Spray column (75 µm i.d. × 50 cm, 2 µm particle size, 100 Å) and separated by elution at a flow rate of 0.25 µL/min at 40 °C by a linear gradient of solvent B (ACN + 0.1% FA) in A, 5% for 3 min, followed by 5% to 20% in 32 min, 20% to 40% in 30 min, 40% to 60% in 20 min and 60% to 98% in 15 min, finishing by holding 98% B 5 min, 98% to 5% in 1 min, and re-equilibrating at 5% B for 20 min. The eluting peptide cations were converted to gas-phase ions by electrospray ionization using a source voltage of 1.75 kV and introduced into the mass spectrometer through a heated ion transfer tube (275 °C). Survey scans of peptide precursors from 200 to 1600 *m*/*z* were performed at 120 K resolution (@ 200 *m*/*z*). Tandem MS was performed by isolation at 1.6 Th with the quadrupole. HCD fragmentation with a normalized collision energy of 35, and rapid scan MS analysis in the ion trap. Only those precursors with charge state 2 ÷ 4 and intensity above the threshold of 5103 were sampled for MS2. The dynamic exclusion duration was set to 60 s with a 10 ppm tolerance around the selected precursor and its isotopes. Monoisotopic precursor selection was turned on. The instrument was run in top speed mode with 3 s cycles, meaning it would continuously perform MS2 events until the list of non-excluded precursors diminished to zero or 3 s, whichever is shorter. MS/MS spectral quality was enhanced enabling the parallelizable time option (i.e., by using all parallelizable time during full scan detection for MS/MS precursor injection and detection). Mass spectrometer calibration was performed by using the Pierce^®^ LTQ Velos ESI Positive Ion Calibration Solution (Thermo Fisher Scientific, Bremen, Germany). MS data acquisition was carried out by utilizing the Xcalibur v. 3.0.63 software (Thermo Fisher Scientific, Bremen, Germany). Three LC-MS/MS replicates for each biological sample were performed.

### 4.4. Database Search and Protein Identification

Protein identification was obtained processing MS data by the PEAKS X de novo sequencing software (Bioinformatics Solutions Inc., Waterloo, ON, Canada). Data were searched against a dedicated protein database, including only the reviewed entries of *Triticum*, *Oryza*, *Hordeum*, *Avena*, *Secale*, *Maize*, and *Brachypodium* species plus the entry of Chicken Lysozyme C (UniProt Acc. No. P00698), downloaded from the UniProt database (release February 2020, 7803 entries). Additionally, the common Repository of Adventitious Proteins (c-RAP) contaminant database (www.thegpm.org, accessed on date 6 February 2020) was included in the database search. 

Database search was carried out using the following parameters: (i) full tryptic peptides with a maximum of 3 missed cleavage sites; (ii) oxidation of methionine, and transformation of N-terminal glutamine and N-terminal glutamic acid residue in the pyroglutamic acid form as variable modifications; (iii) carbamidomethylation of cysteine as a fixed modification. The precursor mass tolerance threshold was 10 ppm and the max fragment mass error was set to 0.6 Da. Peptide Spectral Matches (PSMs) were validated using a Target Decoy PSM Validator node based on q-values at a False Discovery Rate (FDR) ≤ 0.1%. PEAKS score thresholds for PSMs were set to achieve for each database search FDR values for PSMs, Peptide sequences, and Proteins identified below the 0.1% value. A protein was considered identified if a minimum of two unique peptides were matched. Proteins containing the same peptides and that could not be differentiated based on MS/MS analysis alone were grouped to satisfy the principles of parsimony (groups of parsimony). In these cases, proteins from *Triticum*, when identified, were always chosen as the group’s reference protein. When a group of parsimony did not contain a component from *Triticum*, the reference protein was selected from the species closest related to *Triticum* and represented in the group.

### 4.5. Label-Free Quantification

Label-free quantification (LFQ) analysis was performed by the PEAKS Q module, which uses ion peak intensity on MS1. PEAKS Q selects the three most abundant unique peptides for protein quantification by excluding peptides with both modified and unmodified forms and redundant peptides. When a protein is identified with one or two unique peptides only these are used for the quantification. More in detail, the quantification method is based on the detection, separately for each sample, of the peptide features (mass, retention time, and signal intensity) in multiple samples. Then, using the EM (expectation-maximization) algorithm, the features of the same peptide from different samples are aligned together using a high-performance retention time alignment algorithm [52,53]. The following parameters were set for label-free quantification analysis: mass error tolerance (i.e., the mass shift between different runs) 10 ppm; retention time (RT) shift tolerance 3 min (i.e., the maximum elution time range considered for the quantification of an identified peptide between different runs). The only peptide features having the following parameters were considered for the quantification: quality ≥ 7 (a parameter depending on *m*/*z* difference, RT difference, isotope distribution, etc.); average area of the MS signal intensity ≥ 10^5^; peptide charge 2, 3, or 4. A protein was quantified when: (i) at least two of its unique peptides satisfied the parameters above reported; (ii) was identified in a minimum of five out of nine nanoLC-MS/MS runs per flour sample; and (iii) had a *p*-value < 0.05 and a significance ≥ 20 (significance method ANOVA; the significance score was calculated as the −10log10 of the significance testing *p*-value). Finally, in the pairwise comparison, a protein was considered differentially expressed when showed a fold change ≤0.5 or ≥2.

## 5. Conclusions

Over the last few decades, consumers’ perception of food has changed dramatically. Today, foods are intended not only to satisfy the basic human needs of nutrition but also to promote health and prevent diseases. Wheat is one of the most important cereals for mankind, and consumers are increasingly attracted by the old genotypes. This interest is based on the “perceived” higher nutritional value of their flour and their peculiar organoleptic and nutritional features, which are considered of greater quality in comparison with the modern wheat. The old genotypes can be cultivated under environmental-friendly conditions, such as organic farming, and these techniques are considered more “natural” than the techniques used to cultivate modern varieties. Following this process of rediscovery, comparative experiments between old and modern genotypes are desirable to evaluate the real impact of these foods on human health. The quality of grain is related to its protein composition, which may be subjected to change among different growing seasons. Particularly, it depends on the interplay between the genetic characteristics of the plant and external factors (biotic and abiotic) that influence the plant growth such as climatic conditions, chemical and physical characteristics of the soil (substances contained in the soil), and management practices [53] that are usually referred to collectively as the environment.

The present work reports a label-free quantitative comparison in two growing seasons, 2010–2011 and 2011–2012, of the metabolic protein fractions of two old Sicilian landraces, Russello and Timilia, and the modern genotype Simeto that is one of the most widely used commercial cultivars. This study represents the first comprehensive profile of the quantitative composition of the metabolic protein fractions of these two old Sicilian landraces in comparison to a modern cultivar. Pairwise comparisons revealed many proteins whose abundance depends on the growing season rather than the genotype (see Supplementary Materials). On the other hand, also some differentially abundant genotype-related proteins were detected.

The overall results suggest that the old genotype Russello and the modern one Simeto possess a remarkable similarity, with only minor differences. On the contrary, the old genotype Timilia presents major differences in comparison to the other two other genotypes investigated. The different abundance of some stress-related proteins and inhibitors, eventually associated with allergies, suggests that Timilia may adopt a different mechanism of defense in comparison to that of the modern Simeto. Besides, the up-regulation of some proteins involved in seed germination, starch biosynthesis, or playing a positive role in improving the flour mixing characteristics, may reflect some peculiar properties of this genotype (highly resistance to drought and abiotic stresses, the long shelf life of the derived baked products, etc.).

Moreover, the genotype Timilia presents a much higher relative abundance of α-amylase inhibitor 0.28 in comparison with the other two genotypes investigated here. This finding appears very interesting because this protein represents one of the non-gluten components, listed in the Allergome database (http://www.allergome.org/, accessed on date 8 February 2021), likely to be allergenic, and may have a role in nonceliac wheat sensitivity (NCWS) and celiac disease (CD) [54,55,56]. 

Altogether, our data confirm previous results [19,57] leading to the conclusion that from a food and nutritional perspective there is a “substantial equivalence” between old and modern wheat genotypes.

In conclusion, these results may contribute to improving understanding of the relationship between protein profiles of old wheat genotypes and their potential benefits for human consumption. The outcome of this comparison may be useful to improve breeding programs, but also to better understand the relationship between protein profiles of old wheat genotypes and modern ones, their potential benefits for human consumption, and phenotyping properties.

## Figures and Tables

**Table 1:** Protein profile heat map (cell color represents the fold change observed across the genotype investigated). Each cell also reports the corresponding fold change observed in the two growing seasons. Proteins marked with an asterisk were also detected as intra-genotype DAPs. For details see Table 2.

Proteins	Gene Code	Growing Season 2010/2011	Growing Season 2011/2012	Fold Change	
		Fold Change	Fold Change		
Russello vs. Simeto				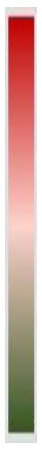	64
Avenin-like b1	AVLB1	**0.12**	**0.12**		
Oil body-associated protein 1A	OBP1A	**4.75**	**9.73**		
Serpin-Z1C	SPZ1C *	**0.4**	**0.21**		
Timilia vs. Simeto					
26 kDa endochitinase 2	CHI2	**0.44**	**0.41**		
a-amylase inhibitor 0.28	IAA2	**64**	**64**		
Antifungal protein R (Fragment)	THHR	**0.27**	**0.32**		
Avenin-like b1	AVLB1 *	**3.33**	**2.5**		
L-ascorbate peroxidase 2 cytosolic	APX2 *	**5.15**	**2.32**		
Oil body-associated protein 1A	OBP1A	**4.6**	**9.09**		
Subtilisin-chymotrypsin inhibitor WSCI	ICIW	**6.61**	**4.67**		1
Sucrose synthase 1	SUS1	**6.31**	**3.95**		
Sucrose synthase 3	SUS3	**3.13**	**2.1**		
Timilia vs. Russello					
26 kD aendochitinase 2	CHI2	**0.27**	**0.33**		
a-amylase inhibitor WDAI-3 (Fragment)	IAA3 *	**0.39**	**0.39**		
Avenin-like b1	AVLB1 *	**38.49**	**25.39**		
Linoleate 9S-lipoxygenase 1	LOX1	**2.42**	**2.28**		
Serpin-Z2A	SPZ2A	**0.02**	**0.02**		
Subtilisin-chymotrypsin inhibitor WSCI	ICIW	**6.74**	**5.81**		
Sucrose synthase 1	SUS1	**3.36**	**2.05**		
Sucrose synthase 3	SUS3	**3.11**	**2.3**		

* proteins marked with an asterisk were also detected as intra-genotype DAPs.

**Table 2:** List of the proteins detected as DAPs in both the growing seasons investigated: Russello vs. Simeto; Timilia vs. Simeto; Timilia vs. Russello. For each protein is reported: Accession Number as used in the Uniprot database; gene code; organism; protein description; the fold change (if ≤0.5 or ≥2); *p*-value observed in the growing seasons 2010–2011 and 2011–2012; significance; the number of the characterized peptides; the number of unique peptides, and the biological process in which it is involved. Proteins that were also detected as intra-genotype DAPs are reported in italics.

UniProt Acc. No.	Gene Code	Organism	Protein Description	Growing Season 2010/2011					Growing Season 2011/2012					Intra-Genotype DAPs *	Biological Process
				Fold Change	*p*-Value	Significance	Peptides	Unique Peptides	Fold Change	*p*-Value	Significance	Peptides	Unique Peptides		
Russello vs. Simeto															
Q2A783	AVLB1	Wheat	Avenin-like b1	0.12	4.84 × 10^−11^	69.40	7	6	0.12	3.03 × 10^−8^	109.29	7	6		Storage
B4FFZ9	OBP1A	Maize	Oil body-associated protein 1A	4.75	7.00 × 10^−5^	64.98	5	5	9.73	3.64 × 10^−14^	88.47	4	4		Others
*Q9ST58*	*SPZ1C*	Wheat	Serpin-Z1C	0.40	1.55 × 10^−7^	48.73	12	5	0.21	1.09 × 10^−8^	105.97	11	3	Y (Russello)	Defense/Inhibitor
Timilia vs. Simeto															
P23951	CHI2	Barley	26 kDa endochitinase 2	0.44	9.95 × 10^−9^	79.42	12	7	0.41	3.01 × 10^−5^	61.90	12	6		Defense/Stress Related Proteins
P01083	IAA2	Wheat	α-amylase inhibitor 0.28	64.00	3.55 × 10^−15^	68.66	4	3	64.00	1.13 × 10^−10^	101.12	6	5		Defense/Inhibitor
P33044	THHR	Barley	Antifungal protein R (Fragment)	0.27	4.72 × 10^−18^	101.88	2	2	0.32	8.77 × 10^−7^	69.64	2	2		Defense/Stress Related Proteins
*Q2A783*	*AVLB1*	Wheat	Avenin-like b1	3.33	3.82 × 10^−9^	95.07	21	21	2.50	7.97 × 10^−6^	55.45	21	21	Y (Timilia)	Storage
*Q9FE01*	*APX2*	Rice	L-ascorbate peroxidase 2 cytosolic	5.15	4.43 × 10^−8^	87.16	2	2	2.32	2.13 × 10^−5^	51.24	2	2	Y (Timilia)	Defense/Stress Related Proteins
B4FFZ9	OBP1A	Maize	Oil body-associated protein 1A	4.60	4.17 × 10^−9^	97.23	5	5	9.09	5.73 × 10^−9^	106.51	4	4		Others
P82977	ICIW	Wheat	Subtilisin-chymotrypsin inhibitor WSCI	6.61	7.87 × 10^−9^	36.55	2	2	4.67	6.87 × 10^−8^	84.71	3	3		Defense/ Inhibitor
P31922	SUS1	Barley	Sucrose synthase 1	6.31	1.37 × 10^−7^	94.78	10	2	3.95	9.51 × 10^−7^	51.48	11	3		Starch biosynthesis
Q43009	SUS3	Rice	Sucrosesynthase 3	3.13	3.21 × 10^−7^	77.26	7	2	2.10	9.56 × 10^−5^	42.12	10	2		Starch biosynthesis
Timilia vs. Russello															
P23951	CHI2	Barley	26 kD aendochitinase 2	0.27	3.29 × 10^−4^	53.12	17	10	0.33	4.27 × 10^−8^	87.68	12	6		Defense/Stress Related Proteins
*P10846*	*IAA3*	Wheat	α-amylase inhibitor WDAI-3 (Fragment)	0.39	1.45 × 10^−3^	36.20	9	4	0.39	3.91 × 10^−6^	33.03	8	3	Y (Timilia)	Defense/Inhibitor
*Q2A783*	*AVLB1*	Wheat	Avenin-like b1	38.49	2.37 × 10^−11^	99.23	8	6	25.39	1.84 × 10^−8^	119.91	8	6	Y (Timilia)	Storage
P29114	LOX1	Barley	Linoleate 9S-lipoxygenase 1	2.42	1.31 × 10^−6^	42.63	8	8	2.28	2.30 × 10^−6^	55.34	9	9		Others
Q9ST57	SPZ2A	Wheat	Serpin-Z2A	0.02	4.04 × 10^−6^	96.72	7	3	0.02	3.47 × 10^−13^	94.42	5	2		Defense/Inhibitor
P82977	ICIW	Wheat	Subtilisin-chymotrypsin inhibitor WSCI	6.74	4.00 × 10^−9^	79.02	2	2	5.81	2.35 × 10^−8^	96.24	3	3		Defense/Inhibitor
P31922	SUS1	Barley	Sucrose synthase 1	3.36	3.11 × 10^−7^	68.34	13	3	2.05	2.74 × 10^−5^	45.72	12	4		Starch biosynthesis
Q43009	SUS3	Rice	Sucrose synthase 3	3.11	6.36 × 10^−7^	58.15	7	2	2.30	1.03 × 10^−5^	50.21	7	2		Starch biosynthesis

* Details are reported in Table S1.

## Data Availability

Not applicable.

## Supplementary Materials

Table S1. List of the proteins differentially abundant in the comparisons between the two growing seasons 2010–11 and 2011–12 for each genotype (intra-genotype DAPs). The growing season 2011–12 was chosen as reference. (a) Comparison *Russello* 2010–11 vs. *Russello* 2011–12. (b) Comparison *Simeto* 2010–11 vs. *Simeto* 2011–12. (c) Comparison *Timilia* 2010–11 vs. *Timilia* 2011–12. For each protein are reported: UniProt Accession Number; description; organism; Significance; the number of peptides; the number of unique peptides; fold-change; *p*-value. Table S2. List of the proteins differentially abundant in the pairwise comparisons among the genotypes investigated for both growing seasons 2010–11 and 2011–12 (inter-genotype DAPs). (a) Comparison *Russello* vs. *Simeto*. (b) Comparison *Timilia* vs. *Simeto*. (c) Comparison *Timilia* vs. *Russello*. In the pairwise comparison of the old genotypes with *Simeto*, the modern cultivar was chosen as reference. In the pairwise comparison between the old genotypes, *Russello* was selected as a reference. For each protein are reported: UniProt Accession Number; description; organism; Significance; the number of peptides; the number of unique peptides; fold-change; p-value; season in which the protein was detected as differentially expressed. Proteins that were also detected as intra-genotype DAPs are reported in italics. Proteins that were detected as differentially abundant in both the growing seasons are reported in bold. Figure S1. Heat maps of the intra-genotypes DAPs identified in the comparison of each genotypes between the two growing seasons. (a) Comparison *Russello* 2010–11 vs. *Russello* 2011–12. (b) Comparison *Simeto* 2010–11 vs. *Simeto* 2011–12. (c) Comparison *Timilia* 2010–11 vs. *Timilia* 2011–12. The growing season 2011–12 was chosen as reference. The relative protein abundance is represented in the map by a color and the map displays trend of each protein in each sample. Over-abundant proteins are in the red zone of the map, instead the under-abundant ones are in the green zone. On the left of the map there is a graph explaining the relationship between these proteins. The hierarchical clustering is generated using a neighbour-joining algorithm with a Euclidean distance similarity measurement of the log_2_ ratios of the abundance of each sample relative to the average abundance. Figure S2. Heat maps of the inter-genotypes DAPs identified in the pairwise comparisons of *Russello vs. Simeto* in both growing seasons, 2010–11 and 2011–12. (a) Comparison *Russello* vs. *Simeto* 2010–11. (b) Comparison *Russello* vs. *Simeto* 2011–12. In the pairwise comparison *Simeto*, the modern cultivar, was chosen as reference. Figure S3. Heat maps of the inter-genotypes DAPs identified in the pairwise comparisons of *Timilia vs. Simeto* in both growing seasons, 2010–11 and 2011–12. (a) Comparison *Timilia* vs. *Simeto* 2010–11. (b) Comparison *Timilia* vs. *Simeto* 2011–12. In the pairwise comparison *Simeto*, the modern cultivar, was chosen as reference. Figure S4. Heat maps of the inter-genotypes DAPs identified in the pairwise comparisons of *Timilia* vs. *Russello* in both growing seasons, 2010–11 and 2011–12. (a) Comparison *Timilia* vs. *Russello* 2010–11. (b) Comparison *Timilia* vs. *Russello* 2011–12. In the pairwise comparison *Russello* was chosen as reference.

## Abbreviations

MS	Mass Spectrometry
CD	Celiac Deasease
IgE	Immunoglobulin E
NCWS	Non Celiac Wheat Sentivity
DAP	Differentially Abundant Proteins
WSCI	Subtilisin Chymotrypsin Inhibitor
ALPs	Avenin-Like Proteins
SUS1	Sucrose Synthase 1
SUS3	Sucrose Synthase 3
LOX	Linoleate Lipoxygenase
FA	Formic Acid
EDTA	Ethylenediaminetetraacetic acid
DTT	Dithiothreitol
IAA	Iodoacetamide
ACN	Acetonitrile
c-RAP	common Repository of Adventitious Proteins
PSMs	Peptide Spectral Matches
FDR	False Discovery Rate
RT	Retention Time
