# Supplementary material for: Quantitative Label-Free Comparison of the Metabolic Protein Fraction in Old and Modern Italian Wheat Genotypes by a Shotgun Approach
Source: Molecules. 2021 Apr 29;26(9):2596. doi: 10.3390/molecules26092596 (PMC8124627; doi:10.3390/molecules26092596)
Supplement: Supplementary file 1 [file molecules-26-02596-s001.zip › molecules-1194414-supplementary.pdf]

Supplementary Material

Quantitative Label-Free Comparison of the Metabolic Protein Fraction in Old and Modern Italian Wheat Genotypes by a Shotgun Approach

Antonella Di Francesco [1], Vincenzo Cunsolo [1,*], Rosaria Saletti [1], Birte Svensson [2], Vera Muccilli [1], Pasquale De Vita [3] and Salvatore Foti [1]

[1] Laboratory of Organic Mass Spectrometry, Department of Chemical Sciences, University of Catania, Viale A. Doria 6, 95125 Catania, Italy
[2] Department of Biotechnology and Bioengineering, Technical University of Denmark, Søltofts Plads, Building 224, 2800 Kgs. Lyngby, Denmark
[3] CREA Research Centre for Cereal and Industrial Crops (CREA-CI), S.S. 673 km 25.200, 71122 Foggia, Italy;

Results

A quantitative label-free comparison of the metabolic protein fractions of two old Sicilian landraces (*Russello* and *Timilia Reste Bianche*) and a modern genotype (*Simeto*) was performed. For each genotype two growing seasons (2010–11 and 2011–12) and three biological replicates for each season were investigated. Moreover, to assess the reproducibility of MS data, each biological replicate was subjected to triplicate RP-nHPLC/nESI-MS/MS analyses, giving rise to eighteen runs for genotype (nine analyses for each season). To detect those proteins whose abundance depends on the growing season (hereinafter called intra-genotype DAPs), each genotype was also investigated by a quantitative comparison between the two seasons 2010–11 and 2011–12. The season 2011–12 was chosen as reference. Then, to identify the differential abundant proteins among the genotype under investigation (hereinafter called inter-genotype DAPs), a pairwise comparison of the three varieties was carried out for each season. In the pairwise comparison of the old genotypes with *Simeto*, the modern cultivar was chosen as reference. In the pairwise comparison between the old genotypes, *Russello* was selected as a reference cultivar.

Intra-genotype DAPs

Each genotype revealed some DAPs (fold-change < 0.5 or > 2.0) between the two growing seasons (hereinafter called intra-genotype DAPs). The list of intra-genotype DAPs is reported in Supplementary Table S1 (a–c).

In *Russello* (Table S1a) 23 DAPs were detected: 21 were up-regulated (i.e., fold-change ≥ 2) and 2 down-regulated (i.e., fold-change ≤ 0.5) in the season 2010–11. The comparison of the two seasons for the modern cultivar *Simeto* (Table S1b) revealed 19 DAPs, all up-regulated in 2010–11. Finally, in *Timilia* (Table S1c) 13 DAPs, all up-regulated in 2010–11, were identified. The corresponding heat maps of intra-genotypes DAPs are reported in Figure S1 (a), (b) and (c), respectively. Heat maps also include some gliadin and glutenin components, due to a partial cross-contamination of metabolic and storage protein fractions. However, gliadins and glutenins were not considered and not inserted in the Tables.

It is interesting to note that in all the three genotypes, many of the DAPs detected between the two seasons present fold-changes near to the threshold. As a consequence, we may conclude that all three genotypes present a very low amount of proteins whose abundance is related with the growing season and the environment (i.e., climatic conditions, chemical and physical characteristics of the soil).

Inter-genotype DAPs

In the Supplementary Table S2 (a–c) are reported the DAPs (fold change ≤ 0.5 or ≥ 2) detected in the pairwise comparison among the genotypes investigated. In particular, Table S2 (a) shows the comparison between *Russello* and *Simeto* for both the seasons

investigated. The results obtained evidence that 8 DAPs were revealed in both season, 2010–11 and 2011–12. The corresponding heat maps are reported in Figure S2 (a) and (b). Most of them are up-regulated in the old cultivar, whereas few proteins appear up-regulated in the modern one. A comparison of the lists of DAPs obtained in the two seasons investigated evidences that ten proteins appear down- or up-regulated only in one out of two seasons. On the contrary, only three proteins show different abundance in both seasons. Particularly, two proteins appear down-regulated in *Russello*, the avenin-like b1 (fold-change 0.12 in both seasons), and the serpin-Z1C (fold-change 0.40 in 2010–11, and 0.21 in 2011–12), whereas the oil body-associated protein 1A is up-regulated (fold-changes 4.75 in 2010-11, and 9.73 in 2011–12). It is important to note that all the DAPs, although with different absolute values, present the same trend in both seasons. Table S2 (b) shows the list of the DAPs detected in the comparison between *Timilia* and *Simeto* for both seasons. The corresponding heat maps are showed in Figure S3 (a) and (b). Sixteen DAPs appear in the season 2010–11, whereas fifteen were detected in the season 2011–12. As reported for the comparison *Russello* vs *Simeto*, most of the DAPs appear up-regulated in the old cultivar. Nine proteins show different abundance in both seasons, whereas 13 proteins are down- or up-regulated only in one out of two seasons. The group of DAPs revealed in both seasons includes two down-regulated components (26 kDa endochitinase 2 and antifungal protein R) and seven up-regulated (α-amylase inhibitor 0.28, avenin-like b1, L-ascorbate peroxidase 2, oil body-associated protein 1A, subtilisin-chymotrypsin inhibitor WSCI, sucrose synthase 1, and sucrose synthase 3). As shown for the comparison *Russello* vs *Simeto*, also in the comparison *Timilia* vs *Simeto* some of the DAPs detected in both seasons present very similar up- or down-regulated trends.

Finally, the results of the comparison of the metabolic fractions extracted from the two old genotypes are reported in Table S2 (c) and Figure S4 (a) and (b). Nineteen proteins are differentially abundant in the season 2010–11, whereas ten DAPs were detected in the season 2011–12. Eight out of the ten DAPs detected in the season 2011–12 were also revealed in the season 2010–11. This group includes 26 kDa endochitinase 2, α-amylase inhibitor WDAI-3, avenin-like b1, linoleate 9S-lipoxygenase 1, serpin-Z2A, subtilisin-chymotrypsin inhibitor WSCI, sucrose synthase 1, and sucrose synthase 3.

Altogether, inter-genotype comparisons evidenced two groups of DAPs: the first group included proteins differentially abundant only in one out of two growing seasons, whereas the second one is constituted by proteins that were differentially abundant in both the growing seasons. The abundance of proteins belonging to the first group cannot be related to the wheat genotype but is probably related to the growing season and the environment. On the contrary, proteins that were differentially abundant in both the growing seasons maybe related to the genotype.

Cross-comparison of inter- and intra-genotypes DAPs

As above reported, the comparison between the two seasons 2010–11 and 2011–12 performed for each genotype revealed that all the genotypes show some proteins whose abundance is related to the growing season (Table S1). On the other hand, the lists of the inter-genotype DAPs detected in the pairwise comparisons between the genotypes for both seasons 2010–11 and 2011–12 (Table S2) reveal that lot of proteins appear down- or up-regulated only in one out of two seasons investigated, whereas others appear differentially abundant in both the growing seasons. Therefore, a cross-comparison between the inter- and intra-genotype DAPs was carried out. This comparison allowed us to ascertain that many of the inter-genotype DAPs were also detected as intra-genotypes for the old landraces (see Table S2). These include some proteins differentially abundant only in one out of two growing seasons (e.g., avenin-like proteins a4 and a6, serpin-Z1A, etc.), but also proteins whose abundance that resulted differentially abundant in both the growing seasons (e.g., serpin Z1-C, avenin-like protein b1, etc.). Besides, several inter-genotype proteins appear differentially abundant only in one out of two seasons but were not detected as intra-genotype DAPs (e.g., 16.9 kDa class I heat shock protein 1, etc.). In the light of these results, all the inter-genotype proteins found as differentially abundant only in

one out of two growing seasons can be hypothesized as depending on the growing season and the environment, rather than intrinsically related to the genotype, and are not discussed in the main manuscript. On the other hand, inter-genotype DAPs in both the growing seasons may be hypothesized genotype-related and are therefore discussed in the main manuscript.

Table S1 list of the proteins differentially abundant in the comparisons between the two growing seasons 2010–11 and 2011–12 for each genotype (intra-genotype DAPs). The growing season 2011–12 was chosen as reference. (a) Comparison *Russello* 2010–11 vs *Russello* 2011–12. (b) Comparison *Simeto* 2010–11 vs *Simeto* 2011–12. (c) Comparison *Timilia* 2010-11 vs *Timilia* 2011–12. For each protein are reported: UniProt Accession Number; description; organism; Significance; the number of peptides; the number of unique peptides; fold-change; *p*-value.

Table S2 list of the proteins differentially abundant in the pairwise comparisons among the genotypes investigated for both growing seasons 2010–11 and 2011–12 (inter-genotype DAPs). (a) Comparison *Russello* vs *Simeto*. (b) Comparison *Timilia* vs *Simeto*. (c) Comparison *Timilia* vs *Russello*. In the pairwise comparison of the old genotypes with *Simeto*, the modern cultivar was chosen as reference. In the pairwise comparison between the old genotypes, *Russello* was selected as a reference. For each protein are reported: UniProt Accession Number; description; organism; Significance; the number of peptides; the number of unique peptides; fold-change; *p*-value; season in which the protein was detected as differentially expressed. Proteins that were also detected as intra-genotype DAPs are reported in italics. Proteins that were detected as differentially abundant in both the growing seasons are reported in bold.

Table 1(a). Russello 2010–11 vs Russello 2011–12.

N.	Accession	Description	Organism	Significance	Peptides	Unique Peptides	Fold Change	*p*-value
1	sp|Q06396|ARF1_ORYSJ	ADP-ribosylation factor 1	Oryza sativa subsp. japonica	31.20	3	3	2.48	8.96×10^{-5}
2	sp|D2KFH1|AVLA4_WHEAT	Avenin-like a4	Triticum aestivum	68.96	35	23	5.08	1.03×10^{-5}
3	sp|P0CZ10|AVLA6_WHEAT	Avenin-like a6	Triticum aestivum	35.77	21	3	2.54	1.15×10^{-3}
4	sp|P00068|CYC_WHEAT	Cytochrome c	Triticum aestivum	32.18	2	2	2.01	1.23×10^{-3}
5	sp|Q03033|EF1A_WHEAT	Elongation factor 1-alpha	Triticum aestivum	39.29	23	5	2.36	5.42×10^{-4}
6	sp|Q7XTH4|GUN11_ORYSJ	Endoglucanase 11	Oryza sativa subsp. japonica	43.63	3	2	2.39	2.65×10^{-4}
7	sp|Q75KH3|GRDH_ORYSJ	Glucose and ribitol dehydrogenase homolog	Oryza sativa subsp. japonica	29.24	5	5	2.08	2.76×10^{-3}
8	sp|P49027|GBLPA_ORYSJ	Guanine nucleotide-binding protein subunit beta-like protein A	Oryza sativa subsp. japonica	46.48	2	2	0.35	7.50×10^{-5}
9	sp|Q41811|H43_MAIZE	Histone H4.3	Zea mays	24.87	2	2	0.47	2.14×10^{-3}
10	sp|P40621|HMGL_WHEAT	HMG1/2-like protein	Triticum aestivum	24.36	2	2	2.70	4.04×10^{-3}
11	sp|Q07661|NDK1_ORYSJ	Nucleotide diphosphate kinase 1	Oryza sativa subsp. japonica	33.08	4	4	2.09	4.79×10^{-3}
12	sp|B4FFK9|OBP2A_MAIZE	Oil body-associated protein 2A	Zea mays	34.91	2	2	2.01	6.25×10^{-4}
13	sp|P12783|PGKY_WHEAT	Phosphoglycerate kinase cytolic	Triticum aestivum	53.33	19	17	3.01	2.52×10^{-4}
14	sp|P0C8Y9|PSA4B_ORYSI	Proteasome subunit alpha type-4-2	Oryza sativa subsp. indica	38.10	2	2	2.49	6.94×10^{-4}
15	sp|Q9LSU3|PSA6_ORYSJ	Proteasome subunit alpha type-6	Oryza sativa subsp. japonica	53.10	3	3	2.08	8.29×10^{-6}
16	sp|P04399|RIP2_HORVU	Protein synthesis inhibitor II	Hordeum vulgare	32.73	8	8	2.11	1.35×10^{-3}

17	sp|P01543|THNB_WHEAT	Purothionin A-1	Triticum aestivum	42.26	8	5	2.61	3.96×10^{-4}
18	sp|Q6AVA8|PPDK1_ORYSJ	Pyruvate phosphate dikinase 1 chloroplastic	Oryza sativa subsp. japonica	69.08	5	2	2.00	1.66×10^{-7}
19	sp|P08819|CBP2_WHEAT	Serine carboxypeptidase 2	Triticum aestivum	26.96	7	3	2.11	5.15×10^{-3}
20	sp|Q41593|SPZ1A_WHEAT	Serpin-Z1A	Triticum aestivum	30.46	23	6	2.14	4.00×10^{-3}
21	sp|Q9ST58|SPZ1C_WHEAT	Serpin-Z1C	Triticum aestivum	20.90	14	4	2.51	1.22×10^{-2}
22	sp|P93692|SPZ2B_WHEAT	Serpin-Z2B	Triticum aestivum	54.24	13	2	4.37	8.42×10^{-5}
23	sp|Q9LDN2|UMPS1_ORYSJ	Uridine 5'-monophosphate synthase	Oryza sativa subsp. japonica	37.48	6	6	2.25	7.15×10^{-4}

Table 1(b). Simeto 2010–11 vs Simeto 2011–12.

N.	Accession	Description	Organism	Significance	Peptides	Unique Peptides	Fold Change	*p*-value
1	sp|Q0JMH0|HACL_ORYSJ	2-hydroxyacyl-CoA lyase	Oryza sativa subsp. japonica	52.88	2	2	2.15	3.69×10^{-7}
2	sp|Q96564|RS27_HORVU	40S ribosomial protein S27	Hordeum vulgare	50.50	3	3	2.45	2.93×10^{-8}
3	sp|P55308|CATA2_HORVU	Catalase isozyme 2	Hordeum vulgare	47.03	7	4	2.02	1.38×10^{-5}
4	sp|P42755|EM4_WHEAT	Em protein H5	Triticum aestivum	24.36	7	3	2.78	1.63×10^{-4}
5	sp|P36183|ENPL_HORVU	Endoplasmin homolog	Hordeum vulgare	61.42	9	7	2.39	1.14×10^{-9}
6	sp|Q75KH3|GRDH_ORYSJ	Glucose and ribitol dehydrogenase homolog	Oryza sativa subsp. japonica	46.77	7	7	2.24	1.06×10^{-8}
7	sp|Q07078|HSP83_ORYSJ	Heat shock protein 81-3	Oryza sativa subsp. japonica	55.59	28	3	2.15	1.67×10^{-6}
8	sp|P40621|HMGL_WHEAT	HMG1/2-like protein	Triticum aestivum	23.31	3	3	5.39	2.07×10^{-2}

9	sp|P82900|NLT2G_WHEAT	Non-specific lipid-transfer protein 2G	Triticum aestivum	46.56	25	5	2.03	1.56×10^{-5}
10	sp|Q9SLZ0|PCKA_MAIZE	Phosphoenolpyruvate carboxykinase (ATP)	Zea mays	42.85	2	2	2.05	3.74×10^{-7}
11	sp|Q84N29|NLTP3_WHEAT	Probable non-specific lipid-transfer protein 3	Triticum aestivum	42.05	3	3	2.34	3.61×10^{-6}
12	sp|Q69LA6|PDX11_ORYSJ	Probable pyridal 5'-phosphate synthase subunit PDX1.1	Oryza sativa subsp. japonica	50.68	4	4	2.04	3.01×10^{-7}
13	sp|P08819|CBP2_WHEAT	Serine carboxypeptidase 2	Triticum aestivum	42.17	9	4	2.11	8.87×10^{-8}
14	sp|Q00445|HS21C_WHEAT	Small heat shock protein chloroplastic	Triticum aestivum	31.81	16	9	2.31	1.04×10^{-3}
15	sp|P55857|SUMO1_ORYSJ	Small ubiquitin-related modifier 1	Oryza sativa subsp. japonica	42.35	2	2	2.23	4.16×10^{-4}
16	sp|P93407|SODCP_ORYSJ	Superoxide dismutase [Cu-Zn] chloroplastic	Oryza sativa subsp. japonica	32.48	4	4	2.03	6.54×10^{-6}
17	sp|O64394|TRXH_WHEAT	Thioredoxin H-type	Triticum aestivum	45.52	16	13	2.22	9.86×10^{-5}
18	sp|Q05806|THN5_WHEAT	Type-5 thionin	Triticum aestivum	44.30	9	9	2.10	1.89×10^{-5}
19	sp|P31251|UBE12_WHEAT	Ubiquitin-activating enzyme E1 2	Triticum aestivum	51.86	5	5	2.04	2.27×10^{-6}

Table 1(c). Timilia 2010–11 vs Timilia 2011–12.

**N.**	**Accession**	**Description**	**Organism**	**Significance**	**Peptides**	**Unique Peptides**	**Fold Change**	***p*-value**
1	sp|Q96564|RS27_HORVU	40S ribosomial protein S27	Hordeum vulgare	51.55	3	3	2.10	2.36×10^{-5}
2	sp|P52894|ALA2_HORVU	Alanine aminotransferase 2	Hordeum vulgare	52.96	8	8	2.22	1.55×10^{-5}
3	sp|P10846|IAA3_WHEAT	Alpha-amylase inhibitor WDAI-3 (Fragment)	Triticum aestivum	45.04	8	3	3.90	1.97×10^{-5}

4	sp|D2KFH1|AVLA4_WHEAT	Avenin-like a4	Triticum aestivum	62.24	21	11	2.83	1.15×10^{-7}
5	sp|Q2A783|AVLB1_WHEAT	Avenin-like b1	Triticum aestivum	57.26	35	35	2.27	3.51×10^{-7}
6	sp|Q7XTH4|GUN11_ORYSJ	Endoglucanase 11	Oryza sativa subsp. japonica	59.13	3	2	2.01	1.11×10^{-7}
7	sp|P40621|HMGL_WHEAT	HMG1/2-like protein	Triticum aestivum	53.00	2	2	2.88	1.27×10^{-4}
8	sp|Q9FE01|APX2_ORYSJ	L-ascorbate peroxidase 2 cytosolic	Oryza sativa subsp. japonica	62.85	3	2	2.80	1.59×10^{-6}
9	sp|P21569|CYPH_MAIZE	Peptidyl-prolyl cis-trans isomerase	Zea mays	20.67	5	5	4.10	7.12×10^{-3}
10	sp|P12783|PGKY_WHEAT	Phosphoglycerate kinase cytosolic	Triticum aestivum	51.09	21	19	2.56	3.34×10^{-7}
11	sp|P52589|PDI_WHEAT	Protein disulfide-isomerase	Triticum aestivum	57.39	42	13	2.18	3.95×10^{-7}
12	sp|Q41593|SPZ1A_WHEAT	Serpin-Z1A	Triticum aestivum	36.48	18	7	2.01	4.93×10^{-5}
13	sp|P93692|SPZ2B_WHEAT	Serpin-Z2B	Triticum aestivum	52.36	2	2	3.23	1.52×10^{-10}

Table 2(a). Russello vs Simeto.

N.	Accession	Description	Significance	Peptides	Unique Peptides	Fold Change	*p*-value	Season	Intra-genotype DAPs
1	sp|P12810|HS16A_WHEAT	16.9 kDa class I heat shock protein 1 OS = Triticum aestivum	47.68	17	11	2.14	4.01×10^{-5}	2011–12	
2	sp|Q9LI00|6PGD1_ORYSJ	6-phosphogluconate dehydrogenase decarboxylating 1 OS = Oryza sativa subsp. japonica	47.83	6	2	2.14	1.29×10^{-6}	2011–12	
3	sp|P01083|IAA2_WHEAT	Alpha-amylase inhibitor 0.28 OS = Triticum aestivum	34.39	3	2	0.17	4.03×10^{-5}	2011–12	
4	sp|P01084|IAA5_WHEAT	Alpha-amylase inhibitor 0.53 OS = Triticum aestivum	22.19	41	18	0.46	3.81×10^{-2}	2010–11	
5	sp|P10846|IAA3_WHEAT	Alpha-amylase inhibitor WDAI-3 (Fragment) OS = Triticum aestivum	39.12	13	8	2.21	1.35×10^{-4}	2011–12	

6	sp|D2KFH1|AVLA4_WHEAT	Avenin-like a4 OS = Triticum aestivum	59.60	24	15	4.98	2.55 × 10^{-5}	2010–11	Y (Russello)
7	sp|P0CZ10|AVLA6_WHEAT	Avenin-like a6 OS = Triticum aestivum	31.62	15	2	2.64	2.10 × 10^{-3}	2010–11	Y (Russello)
8	sp|Q2A783|AVLB1_WHEAT	Avenin-like b1 OS=Triticum aestivum	69.40	7	6	0.12	4.84 × 10^{-11}	2010–11	
	sp|Q2A783|AVLB1_WHEAT	Avenin-like b1 OS = Triticum aestivum	109.29	7	6	0.12	3.03 × 10^{-8}	2011–12	
9	sp|P30110|GSTF1_WHEAT	Glutathione S-transferase 1 OS = Triticum aestivum	63.93	4	4	2.94	4.14 × 10^{-7}	2011–12	
10	sp|B4FFZ9|OBP1A_MAIZE	Oil body-associated protein 1A OS = Zea mays	64.98	5	5	4.75	7.00 × 10^{-5}	2010–11	
	sp|B4FFZ9|OBP1A_MAIZE	Oil body-associated protein 1A OS = Zea mays	88.47	4	4	9.73	3.64 × 10^{-14}	2011–12	
11	sp|Q41593|SPZ1A_WHEAT	Serpin-Z1A OS = Triticum aestivum	35.40	17	6	2.53	1.50 × 10^{-3}	2010–11	Y (Russello)
12	sp|Q9ST58|SPZ1C_WHEAT	Serpin-Z1C OS = Triticum aestivum	48.73	12	5	0.40	1.55 × 10^{-7}	2010–11	Y (Russello)
	sp|Q9ST58|SPZ1C_WHEAT	Serpin-Z1C OS = Triticum aestivum	105.97	11	3	0.21	1.09 × 10^{-8}	2011–12	
13	sp|Q9ST57|SPZ2A_WHEAT	Serpin-Z2A OS = Triticum aestivum	45.77	22	14	3.47	6.55 × 10^{-4}	2010–11	

Table 2(b). Timilia vs Simeto.

N.	Accession	Description	Significance	Peptides	Unique Peptides	Fold Change	*p*-value	Season	Intra-genotype DAPs
1	sp|Q43470|1433B_HORVU	14-3-3-like protein B OS = Hordeum vulgare	27.47	12	3	2.18	2.64 × 10^{-5}	2010–11	
2	sp|Q6W8Q2|REHY_WHEAT	1-Cys peroxiredoxin PER1 OS = Triticum aestivum	24.75	32	5	0.39	1.10 × 10^{-2}	2011–12	

3	sp|P23951|CHI2_HORVU	26 kDa endochitinase 2 OS = Hordeum vulgare	79.42	12	7	0.44	9.95×10^{-9}	2010–11	
	sp|P23951|CHI2_HORVU	26 kDa endochitinase 2 OS = Hordeum vulgare	61.90	12	6	0.41	3.01×10^{-5}	2011–12	
4	sp|O24396|PURA_WHEAT	Adenylosuccinate synthetase chloroplastic (Fragment) OS = Triticum aestivum	25.59	2	2	2.21	9.81×10^{-4}	2011–12	
5	sp|P01083|IAA2_WHEAT	Alpha-amylase inhibitor 0.28 OS = Triticum aestivum	68.66	4	3	64.00	3.55×10^{-15}	2010–11	
	sp|P01083|IAA2_WHEAT	Alpha-amylase inhibitor 0.28 OS = Triticum aestivum	101.12	6	5	64.00	1.13×10^{-10}	2011–12	
6	sp|P10846|IAA3_WHEAT	Alpha-amylase inhibitor WDAI-3 (Fragment) OS = Triticum aestivum	40.04	9	4	2.50	2.70×10^{-4}	2010–11	Y (Timilia)
7	sp|P33044|THHR_HORVU	Antifungal protein R (Fragment) OS = Hordeum vulgare	101.88	2	2	0.27	4.72×10^{-18}	2010–11	
	sp|P33044|THHR_HORVU	Antifungal protein R (Fragment) OS = Hordeum vulgare	69.64	2	2	0.32	8.77×10^{-7}	2011–12	
8	sp|P37833|AATC_ORYSJ	Aspartate aminotransferase cytoplasmic OS = Oryza sativa subsp. japonica	38.90	9	9	2.01	1.65×10^{-2}	2011–12	
9	sp|D2KFH1|AVLA4_WHEAT	Avenin-like a4 OS = Triticum aestivum	75.31	21	9	2.67	3.13×10^{-8}	2010–11	Y (Timilia)
10	sp|Q2A783|AVLB1_WHEAT	Avenin-like b1 OS = Triticum aestivum	95.07	21	21	3.33	3.82×10^{-9}	2010–11	Y (Timilia)
	sp|Q2A783|AVLB1_WHEAT	Avenin-like b1 OS = Triticum aestivum	55.45	21	21	2.50	7.97×10^{-6}	2011–12	
11	sp|Q9FRV0|CHIC_SECCE	Basic endochitinase C OS = Secale cereale	83.62	10	4	2.41	3.01×10^{-7}	2010–11	
12	sp|P49027|GBLPA_ORYSJ	Guanine nucleotide-binding protein subunit beta-like protein A OS = Oryza sativa subsp. japonica	49.78	3	3	2.71	1.27×10^{-6}	2011–12	
13	sp|Q9FE01|APX2_ORYSJ	*L*-ascorbate peroxidase 2 cytosolic OS = Oryza sativa subsp. japonica	87.16	2	2	5.15	4.43×10^{-8}	2010–11	Y (Timilia)
	sp|Q9FE01|APX2_ORYSJ	*L*-ascorbate peroxidase 2 cytosolic OS=Oryza sativa subsp. japonica	51.24	2	2	2.32	2.13×10^{-5}	2011–12	
14	sp|P24296|NLTP1_WHEAT	Non-specific lipid-transfer protein (Fragment) OS = Triticum aestivum	42.39	21	21	0.50	1.94×10^{-4}	2011–12	
15	sp|B4FFZ9|OBP1A_MAIZE	Oil body-associated protein 1A OS = Zea mays	97.23	5	5	4.60	4.17×10^{-9}	2010–11	

N.	Accession	Description	Significance	Peptides	Unique Peptides	Fold Change	p-value	Season	Intra-genotype DAPs
	sp|B4FFZ9|OBP1A_MAIZE	Oil body-associated protein 1A OS = Zea mays	106.51	4	4	9.09	5.73×10^{-9}	2011–12	
16	sp|P12783|PGKY_WHEAT	Phosphoglycerate kinase cytosolic OS = Triticum aestivum	51.44	22	20	2.53	4.30×10^{-7}	2010–11	Y (Timilia)
17	sp|Q41593|SPZ1A_WHEAT	Serpin-Z1A OS = Triticum aestivum	85.87	16	6	2.87	3.30×10^{-8}	2010–11	Y (Timilia)
18	sp|Q9ST58|SPZ1C_WHEAT	Serpin-Z1C OS = Triticum aestivum	44.54	11	6	0.35	4.22×10^{-6}	2011–12	
19	sp|P82977|ICIW_WHEAT	Subtilisin-chymotrypsin inhibitor WSCI OS = Triticum aestivum	36.55	2	2	6.61	7.87×10^{-9}	2010–11	
	sp|P82977|ICIW_WHEAT	Subtilisin-chymotrypsin inhibitor WSCI OS = Triticum aestivum	84.71	3	3	4.67	6.87×10^{-8}	2011–12	
20	sp|P31922|SUS1_HORVU	Sucrose synthase 1 OS = Hordeum vulgare	94.78	10	2	6.31	1.37×10^{-7}	2010–11	
	sp|P31922|SUS1_HORVU	Sucrose synthase 1 OS = Hordeum vulgare	51.48	11	3	3.95	9.51×10^{-7}	2011–12	
21	sp|P31923|SUS2_HORVU	Sucrose synthase 2 OS = Hordeum vulgare	69.71	17	10	3.60	1.68×10^{-7}	2010–11	
22	sp|Q43009|SUS3_ORYSJ	Sucrose synthase 3 OS = Oryza sativa subsp. Japonica	77.26	7	2	3.13	3.21×10^{-7}	2010–11	
	sp|Q43009|SUS3_ORYSJ	Sucrose synthase 3 OS = Oryza sativa subsp. japonica	42.12	10	2	2.10	9.56×10^{-5}	2011–12	

Table 2(c). Timilia vs Russello.

N.	Accession	Description	Significance	Peptides	Unique Peptides	Fold Change	*p*-value	Season	Intra-genotype DAPs
1	sp|P23951|CHI2_HORVU	26 kDa endochitinase 2 OS = Hordeum vulgare	53.12	17	10	0.27	3.29×10^{-4}	2010–11	
	sp|P23951|CHI2_HORVU	26 kDa endochitinase 2 OS = Hordeum vulgare	87.68	12	6	0.33	4.27×10^{-8}	2011–12	
2	sp|P49210|RL9_ORYSJ	60S ribosomial protein L9 OS = Oryza sativa subsp. Japonica	75.66	2	2	2.53	2.74×10^{-8}	2010–11	

3	sp|P51823|ARF2_ORYSJ	ADP-ribosylation factor 2 OS = Oryza sativa subsp. Japonica	28.42	3	3	2.23	5.59×10^{-5}	2011–12	
4	sp|P01083|IAA2_WHEAT	Alpha-amylase inhibitor 0.28 OS = Triticum aestivum	77.05	3	2	64.00	7.96×10^{-11}	2011–12	
5	sp|P10846|IAA3_WHEAT	Alpha-amylase inhibitor WDAI-3 (Fragment) OS = Triticum aestivum	36.20	9	4	0.39	1.45×10^{-3}	2010–11	Y (Timilia)
	sp|P10846|IAA3_WHEAT	Alpha-amylase inhibitor WDAI-3 (Fragment) OS = Triticum aestivum	33.03	8	3	0.39	3.91×10^{-6}	2011–12	
6	sp|P33044|THHR_HORVU	Antifungal protein R (Fragment) OS = Hordeum vulgare	40.19	3	3	0.50	2.72×10^{-4}	2010–11	
7	sp|D2KFH1|AVLA4_WHEAT	Avenin-like a4 OS = Triticum aestivum	51.03	30	16	0.38	1.55×10^{-4}	2010–11	Y (Timilia; Russello)
8	sp|P0CZ11|AVLA7_WHEAT	Avenin-like a7 OS = Triticum aestivum	45.66	18	3	0.47	3.33×10^{-7}	2010–11	
9	sp|Q2A783|AVLB1_WHEAT	Avenin-like b1 OS = Triticum aestivum	99.23	8	6	38.49	2.37×10^{-11}	2010–11	Y (Timilia)
	sp|Q2A783|AVLB1_WHEAT	Avenin-like b1 OS = Triticum aestivum	119.91	8	6	25.39	1.84×10^{-8}	2011–12	
10	sp|P49027|GBLPA_ORYSJ	Guanine nucleotide-binding protein subunit beta-like protein A OS = Oryza sativa subsp. Japonica	78.13	3	3	2.91	1.79×10^{-7}	2010–11	Y (Russello)
11	sp|A2WKP5|H2B4_ORYSI	Histone H2B.4 OS = Oryza sativa subsp. Indica	45.33	2	2	2.17	3.95×10^{-6}	2010–11	
12	sp|Q9FE01|APX2_ORYSJ	*L*-ascorbate peroxidase 2 cytosolic OS = Oryza sativa subsp. Japonica	64.96	2	2	4.86	9.10×10^{-8}	2010–11	Y (Timilia)
13	sp|P29114|LOX1_HORVU	Linoleate 9S-lipoxygenase 1 OS = Hordeum vulgare	42.63	8	8	2.42	1.31×10^{-6}	2010–11	
	sp|P29114|LOX1_HORVU	Linoleate 9S-lipoxygenase 1 OS = Hordeum vulgare	55.34	9	9	2.28	2.30×10^{-6}	2011–12	
14	sp|P04399|RIP2_HORVU	Protein synthesis inhibitor II OS = Hordeum vulgare	35.87	7	7	0.46	1.45×10^{-3}	2010–11	Y (Russello)
15	sp|Q9ST57|SPZ2A_WHEAT	Serpin-Z2A OS = Triticum aestivum	96.72	7	3	0.02	4.04×10^{-6}	2010–11	

	sp|Q9ST57|SPZ2A_WHEAT	Serpin-Z2A OS = Triticum aestivum	94.42	5	2	0.02	3.47×10^{-13}	2011-12	
16	sp|P93692|SPZ2B_WHEAT	Serpin-Z2B OS = Triticum aestivum	33.66	7	3	0.41	1.88×10^{-3}	2010-11	Y (Timilia; Russello)
17	sp|P82977|ICIW_WHEAT	Subtilisin-chymotrypsin inhibitor WSCI OS = Triticum aestivum	79.02	2	2	6.74	4.00×10^{-9}	2010–11	
	sp|P82977|ICIW_WHEAT	Subtilisin-chymotrypsin inhibitor WSCI OS = Triticum aestivum	96.24	3	3	5.81	2.35×10^{-8}	2011–12	
18	sp|P31922|SUS1_HORVU	Sucrose synthase 1 OS = Hordeum vulgare	68.34	13	3	3.36	3.11×10^{-7}	2010–11	
	sp|P31922|SUS1_HORVU	Sucrose synthase 1 OS = Hordeum vulgare	45.72	12	4	2.05	2.74×10^{-5}	2011–12	
19	sp|P31923|SUS2_HORVU	Sucrose synthase 2 OS = Hordeum vulgare	41.01	15	8	3.27	1.02×10^{-6}	2010–11	
20	sp|Q43009|SUS3_ORYSJ	Sucrose synthase 3 OS = Oryza sativa subsp. Japonica	58.15	7	2	3.11	6.36×10^{-7}	2010–11	
	sp|Q43009|SUS3_ORYSJ	Sucrose synthase 3 OS = Oryza sativa subsp. japonica	50.21	7	2	2.30	1.03×10^{-5}	2011–12	
21	sp|Q94DM8|UFM1_ORYSJ	Ubiquitin-fold modifier 1 OS = Oryza sativa subsp. Japonica	35.51	2	2	2.09	1.92×10^{-4}	2010–11	

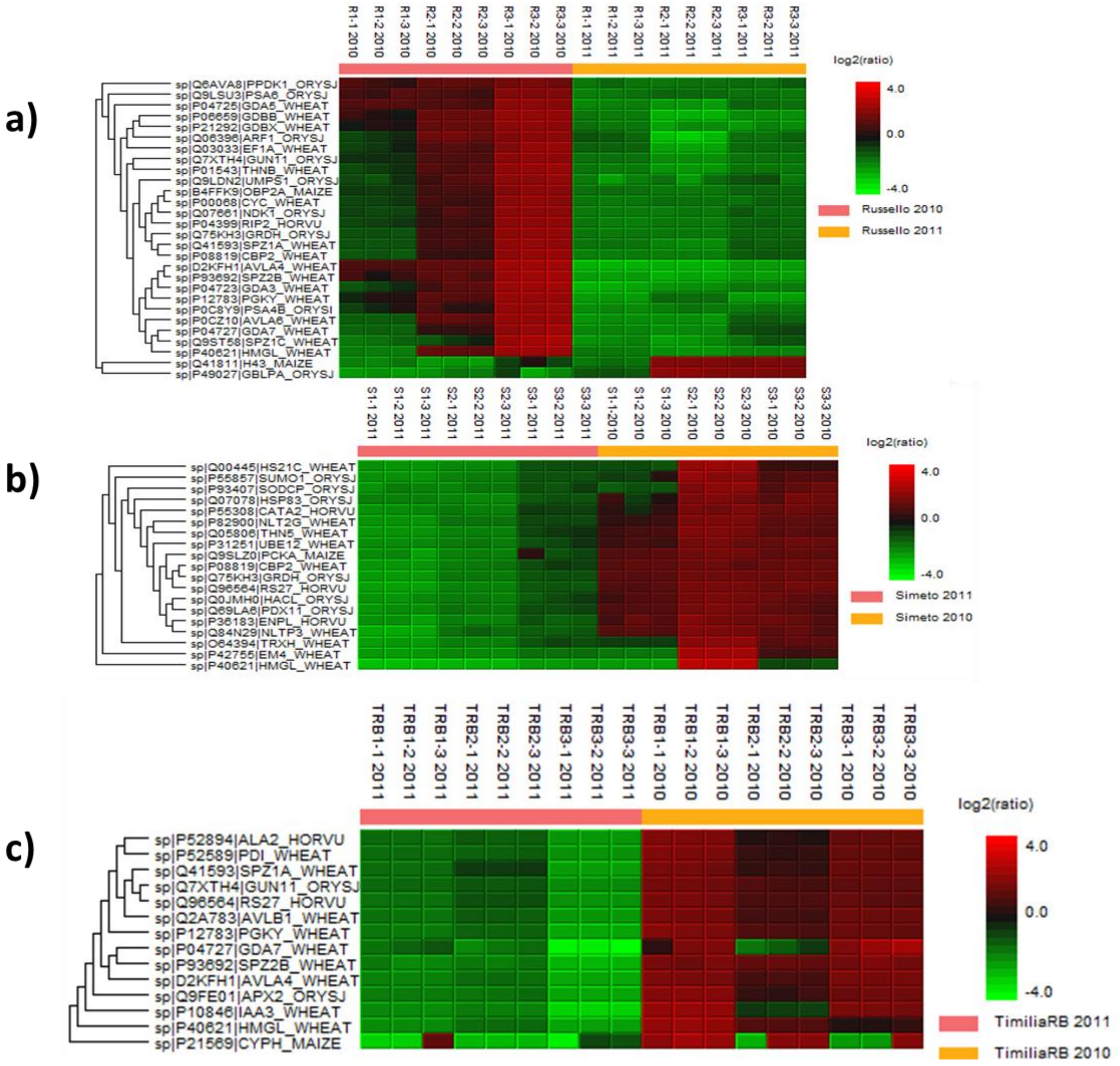

Figure S1. Heat maps of the intra-genotypes DAPs identified in the comparison of each genotypes between the two growing seasons. (**a**) Comparison *Russello* 2010–11 vs *Russello* 2011–12. (**b**) Comparison *Simeto* 2010–11 vs *Simeto* 2011–12. (**c**) Comparison *Timilia* 2010–11 vs *Timilia* 2011–12. The growing season 2011–12 was chosen as reference. The relative protein abundance is represented in the map by a color and the map displays trend of each protein in each sample. Over-abundant proteins are in the red zone of the map, instead the under-abundant ones are in the green zone. On the left of the map there is a graph explaining the relationship between these proteins. The hierarchical clustering is generated using a neighbour-joining algorithm with a Euclidean distance similarity measurement of the $\log_2$ ratios of the abundance of each sample relative to the average abundance.

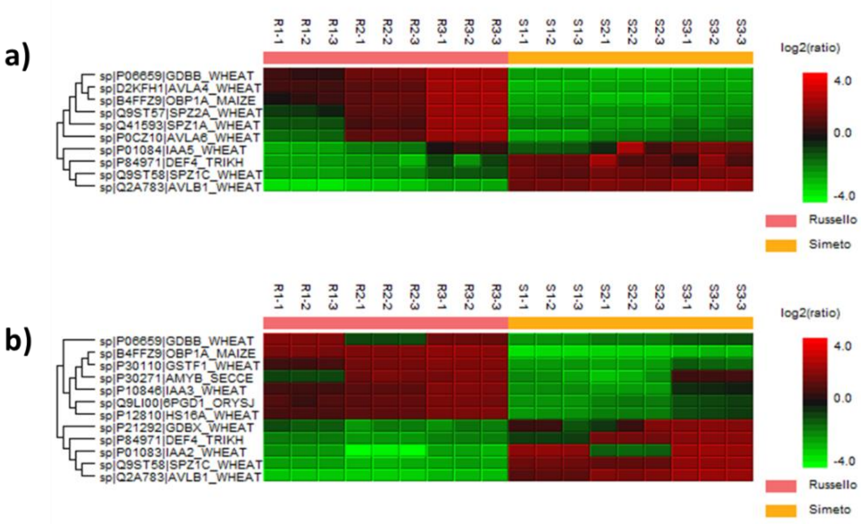

Figure S2. Heat maps of the inter-genotypes DAPs identified in the pairwise comparisons of *Russello vs Simeto* in both growing seasons, 2010–11 and 2011–12. (**a**) Comparison *Russello* vs *Simeto* 2010–11. (**b**) Comparison *Russello* vs *Simeto* 2011–12. In the pairwise comparison *Simeto*, the modern cultivar, was chosen as reference.

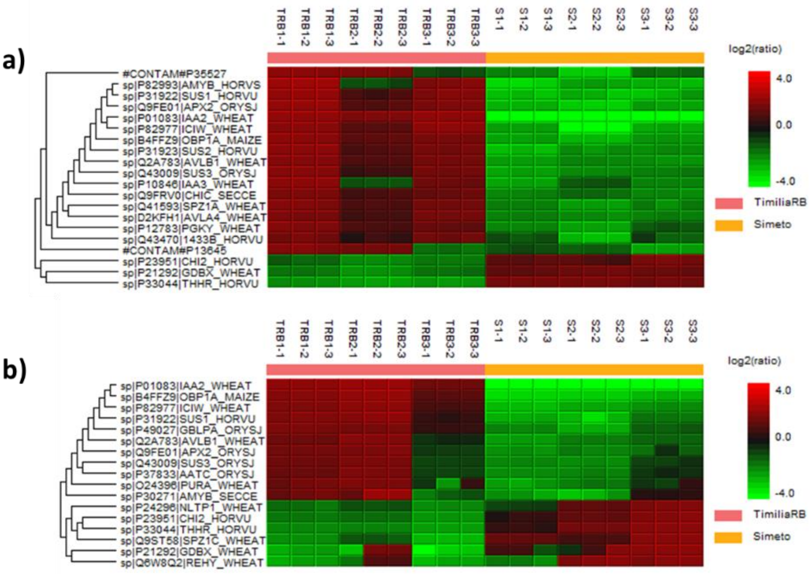

Figure S3. Heat maps of the inter-genotypes DAPs identified in the pairwise comparisons of *Timilia vs Simeto* in both growing seasons, 2010–11 and 2011–12. (**a**) Comparison *Timilia* vs *Simeto* 2010–11. (**b**) Comparison *Timilia* vs *Simeto* 2011–12. In the pairwise comparison *Simeto*, the modern cultivar, was chosen as reference.

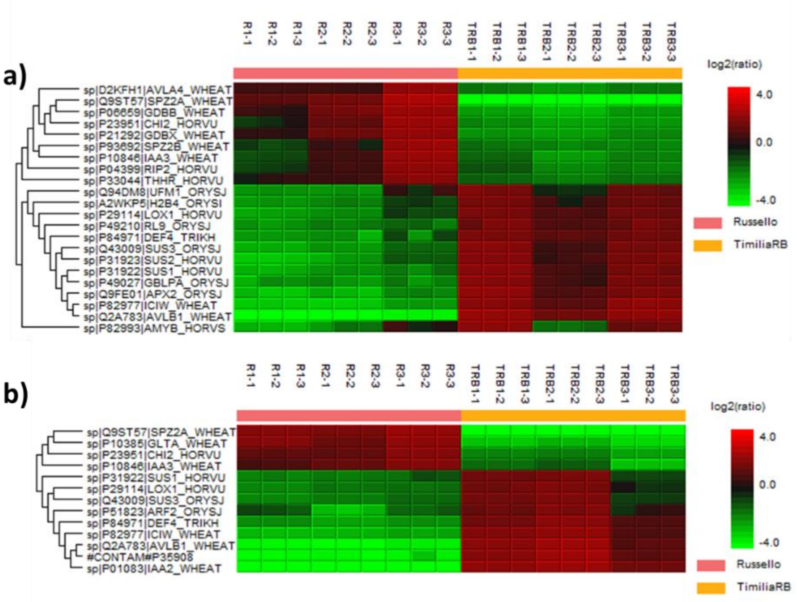

Figure S4. Heat maps of the inter-genotypes DAPs identified in the pairwise comparisons of *Timilia vs Russello* in both growing seasons, 2010–11 and 2011–12. (**a**) Comparison *Timilia* vs *Russello* 2010–11. (**b**) Comparison *Timilia* vs *Russello* 2011–12. In the pairwise comparison *Russello* was chosen as reference.
